# Printable Nanomaterials for the Fabrication of High-Performance Supercapacitors

**DOI:** 10.3390/nano8070528

**Published:** 2018-07-13

**Authors:** Jiazhen Sun, Bo Cui, Fuqiang Chu, Chenghu Yun, Min He, Lihong Li, Yanlin Song

**Affiliations:** 1 Key Laboratory of Pulp, Paper, Printing & Packaging of China National Light Industry, Key Laboratory of Printing & Packaging Materials and Technology of Shandong Province, School of Printing and Packaging Engineering, Qilu University of Technology (Shandong Academy of Sciences), Jinan 250353, China; jiazhensun@qlu.edu.cn (J.S.); cuibo@qlu.edu.cn (B.C.); fqchu@126.com (F.C.); chenghuyun@qlu.edu.cn (C.Y.); 2 Key Laboratory of Green Printing, Beijing Engineering Research Center of Nanomaterials for Green Printing Technology, Beijing National Laboratory for Molecular Sciences, Institute of Chemistry, Chinese Academy of Sciences, Beijing 100190, China; heminyiwen@iccas.ac.cn (M.H.); lilihong1209@iccas.ac.cn (L.L.)

**Keywords:** supercapacitors, printing techniques, nanomaterials, patterning, structure

## Abstract

In recent years, supercapacitors are attracting great attention as one kind of electrochemical energy storage device, which have a high power density, a high energy density, fast charging and discharging, and a long cycle life. As a solution processing method, printing technology is widely used to fabricate supercapacitors. Printable nanomaterials are critical to the fabrication of high-performance supercapacitors by printing technology. In this work, the advantages of printing technology are summarized. Moreover, various nanomaterials used to fabricate supercapacitors by printing technology are presented. Finally, the remaining challenges and broad research as well as application prospects in printing high-performance supercapacitors with nanomaterials are proposed.

## 1. Introduction

Electrochemical energy storage systems play an important role in various applications, such as electric vehicles, electronics, and illuminators [1,2,3]. Systems for electrochemical energy storage include batteries and supercapacitors. In the batteries, electrical energy is stored in batteries via redox reactions at the anode and cathode, which induces a volumetric change of electrochemical energy in the storage electrode in charging and discharging. This volumetric change would produce a limited lifetime, usually only hundreds of cycles. The redox reactions at electrode in battery also provide a low power density, which restricts the applications in many occasions [4,5,6]. In comparison to batteries, supercapacitors are attracting great attention as one kind of electrochemical energy storage device, which have a high power density, a high energy density, fast charging and discharging, and a long cycle life. Supercapacitors have a balance of power density and energy density between the batteries and the capacitors. For the supercapacitors, electrical energy is delivered by movement of the electrolyte ions at the electrolyte/electrode interface, and a parallel movement of electrons occurs in the external wire. The mechanism of supercapacitors determines that there is no volumetric change of electrochemical energy in the storage electrode in charging and discharging. Meanwhile, a high power density, a high energy density, fast charging and discharging, and a long cycle life are obtained by using supercapacitors as the electrochemical energy storage system. Supercapacitors are usually composed of negative electrodes, positive electrodes, and separators that are soaked with electrolytes. The mechanism and structure of supercapacitors also determine the performance of the supercapacitor, which could be used to improve many special areas of application by synthesizing materials and designing structures [7,8]. Currently, many kinds of supercapacitors such as the stretchable supercapacitor, editable supercapacitor, self-healing supercapacitor, micro supercapacitors, and fiber shaped supercapacitor are developed for using in displays, sensors, flexible electronics, portable electronics, wearable electronics, and so on [9,10].

Printing technology as a solution processing method, which could realize a high throughput of functional patterns with a precise structure on nearly any substrate, is widely used to fabricate high-performance supercapacitors [11,12,13]. Printing techniques including offset printing, gravure printing, flexographic printing, screen printing, micro-contact printing, transfer printing, inkjet printing, extrusion printing and spray printing could be used to realize various micro-nano structures with functional inks. In order to precisely design the printed structure, the feature and principle of the printing process should be mastered [14]. Meanwhile, the printability of functional ink also influences the printed structures [15]. In addition, the depositing behavior of printed ink plays a crucial role in controlling the depositing structures of functional printing materials [16,17,18]. Therefore, the printed micro-nano structures could provide a low-cost, convenient, flexible, scalable, and speedy way to fabricate various high-performance supercapacitors with the proper functional ink [19,20].

Recently, nanomaterials have aroused great attention due to their broad applications [21,22,23,24]. Nanomaterials are used to fabricate the active electrode of supercapacitors, which could promote the performance of electric double-layer capacitors, pseudocapacitors, and hybrid capacitors. The research and development of nanomaterials have greatly improved the performance of supercapacitors. Based on design and preparation of printable nanomaterials, high-performance supercapacitors have been developed by printing technology [25,26,27]. The research of carbon nanomaterial inks is expected to achieve a high electric double layer capacitor performance. Metal oxide nanomaterial and conductive polymer could be applied as inks to print pseudocapacitors directly, which significantly promote pseudocapacitor performance [28,29]. Over all, printable nanomaterials have shown promising prospects in fabricating high-performance supercapacitors, and will lead the printed supercapacitor into a new age of portability and flexibility [30,31].

In this work, the advantages of printing technology are summarized. Moreover, various nanomaterials used to fabricate supercapacitors by printing technology are presented. Finally, the remaining challenges and the broad research as well as application prospects in printing high-performance supercapacitors with nanomaterials are proposed.

## 2. Advances of Printing Technology

In general, printing technology could carry the colorful ink to the substrate, and then the graphic information could be expressed and reproduced with high resolution and speediness. This process makes a great contribution to the publishing and packaging. Meanwhile, printing technology is also deemed as a micro-nano manufacturing method in fabricating various photoelectric devices or 3D structures. When functional materials are prepared into inks with proper printabilities, the functional material patterning could be realized with different printing techniques, such as offset printing, gravure printing, flexographic printing, screen printing, micro-contact printing, transfer printing, inkjet printing, extrusion printing, and spray printing. In the following, the advantages of printing technology are summarized with plate-based printing techniques and digital printing techniques.

### 2.1. Plate-Based Printing Techniques

**Figure 1:**
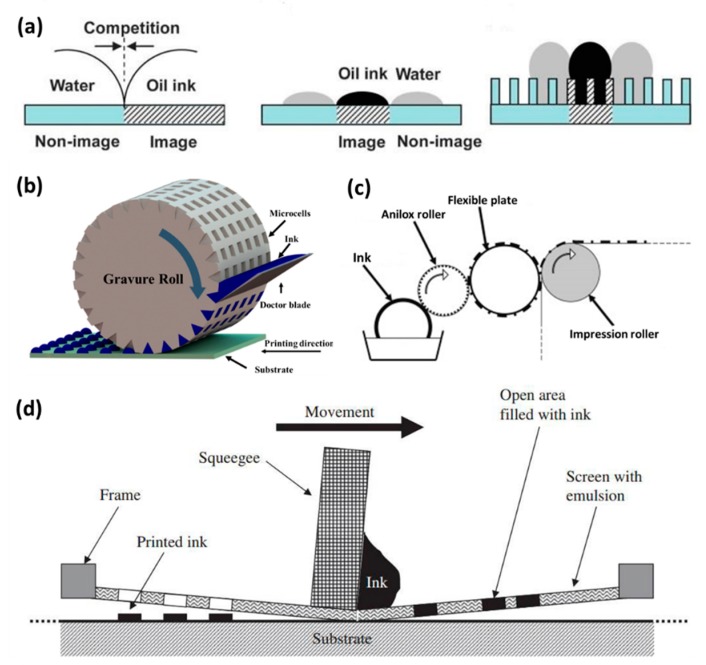
(**a**) The scheme is the role of plate for controlling ink patterning in offset printing technique. The hydrophilic area is the non-image area, and the other area is the image area. The effect of surface energy and surface structure on the wetting behavior of oil ink and water are exhibited. The water is on the hydrophilic and non-image area which could repel the oil ink. The plate is the chemically patterned flat plate. The plate is the chemically and structured patterned plate. Reproduced from [33], with permission from Royal Society of Chemistry, 2013; (**b**) The processing steps of gravure printing: ink filling in the microcells, the doctor blade wiping the ink, ink transferring to the substrate, the functional material patterning formation. Reproduced from [35], with permission from American Chemical Society, 2014; (**c**) The processing steps of flexographic printing: ink transferring to the anilox roller, ink transferring to the flexible plate, ink transferring to the substrate with the impression roller; (**d**) The processing steps of screen printing. The ink is squeezed through the pores from the mesh plate with the auto device, and then the functional ink is deposited onto the required area of printing substrate. Reproduced from [40], with permission from Elsevier, 2009.

The wettability between ink and printing plate could be used to adjust the printing structures within plate-based printing techniques. There are four traditional plate-based printing techniques: offset printing, gravure printing, flexographic printing, screen printing. Offset printing plays an important role in high-resolution image or text printing. The printing plate in offset printing has a patterned surface energy, which could realize the ink’s adhesion or repellency [32]. The rheology of offset printing ink requires the ink has a proper thixotropism and shear thinning. The viscosity of the ink is 5–200 Pa·S. The printing speed of the technique is 15–1000 m/min. The printed resolution is 20–50 µm. The printed thickness is 0.5–2 µm. The surface energy is applied to realize the ink patterning in the offset printing. The typical way is the water in the hydrophilic and the non-image area and then the oil ink in the image area. The application of surface energy for patterning functional material is shown in Figure 1a, the chemical and structural surface properties of printing plate directly influence the patterning of ink [33]. Meanwhile, waterless offset printing is also developed to pattern functional materials with a low surface energy area to repel the oil ink [34]. The gravure printing technique uses a plate with microcells to pattern ink. In the printing process, the blade is used to remove the ink on the surface of plate, and then only the ink in microcells is transferred to the substrate. The viscosity of the ink is 50–200 mPa·S. The printing speed of the technique is 20–1000 m/min. The printed resolution is 20–75 µm. The printed thickness is 0.1–5 µm. The gravure printing could realize a gorgeous product with a speedy fabrication. The printing process is presented in Figure 1b [35]. The inverse printing process is also developed to pattern conductive ink [36]. The doctor blade could also be removed with a large wettability contrast [37]. The flexographic printing technique uses a flexible plate to pattern ink. In the printing process, the engraved cells transfer homogeneous ink layer on the raised area of flexible plate, and then the ink layer on the raised area is transferred to the printing substrate [38]. The viscosity of the ink is 50–500 mPa·S. The printing speed of the technique is 50–500 m/min. The printed resolution is 30–75 µm. The printed thickness is 0.5–8 µm. The flexographic printing commonly uses water-based ink and a paper substrate, which could realize the aim of environmental protection. The printing process is shown in Figure 1c. Once the flexible plate has a proper scale, a dense ink structure could be generated with the same scale on the surface of the substrate. Flexographic printing has been used to produce a radio frequency identification (RFID) circuit for electric tags with conductive ink [39]. Screen printing is a unique printing technique with a mesh plate, which plays an important role in patterning functional materials. As shown in Figure 1d, the ink is squeezed through the pores from the mesh plate, and then the ink is deposited onto the substrate [40]. The viscosity of the ink is 500–50,000 mPa·S. The printing speed of the technique is 10–100 m/min. The printed resolution is 50–100 µm. The printed thickness is 3–100 µm. As a way of fabricating with a thick ink layer, screen printing could be used to print marks, logos, and billboards. In addition, screen printing has been used to make conductive circuits of solar cells, touch panels, and field effect transistors. Lots of studies have been done in developing conductive ink for screen printing, which includes the metal nanoparticle ink, metal nanowire ink, and carbon ink [41,42]. Meanwhile, the printing technique is developed for a roll-to-roll fabricating process, which enables improvement of the producing scale. The printing resolution could also be improved with the proper density of the mesh and rheology of the ink.

**Figure 2:**
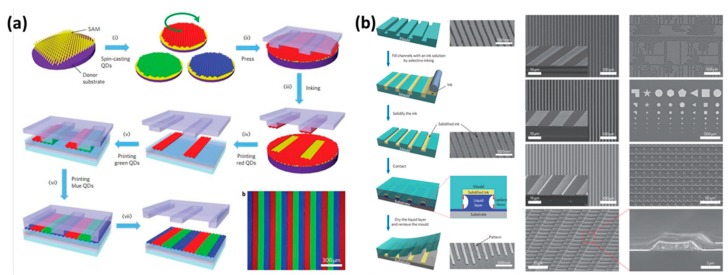
(**a**) The processing steps of micro-contact printing. Functional materials on the modified surface could be adhered to the printing stamp, and then the functional materials are deposited on specific regions of surfaces. Functional material patterning formation. Reproduced from [44], with permission from Macmillan Publishers Ltd., 2011; (**b**) The processing steps of transfer printing. Left: Ink filling in the templates and transferring with a high-resolution. Right: Patterned functional micro-nano structure. Reproduced from [45], with permission from Macmillan Publishers Ltd., 2011.

As the interactions between ink and substrate could be adjusted with special energy, such as covalent bonds, electrostatic interaction, or physical adhesion, micro-contact printing has been successfully applied to pattern many materials, such as organic molecules, polymers, proteins, nanoparticles, colloids, and metals [43]. As shown in Figure 2a, functional materials on the modified surface could be adhered to the printing stamp, and then the functional materials are deposited on specific regions of surfaces. The micro-contact printing provides a facile way to pattern various functional materials [44]. In order to precisely control the material positioning, many kinds of transfer printing have been developed through a physicochemical interacting template. Ink could be restricted or dewetted to a micro-nano structure with the template (Figure 2b) [45]. In practical applications, there are many special transfer printing techniques based on physicochemical interaction, such as thermal transfer printing, static-electricity, and water transfer printing.

### 2.2. Digital Printing Techniques

**Figure 3:**
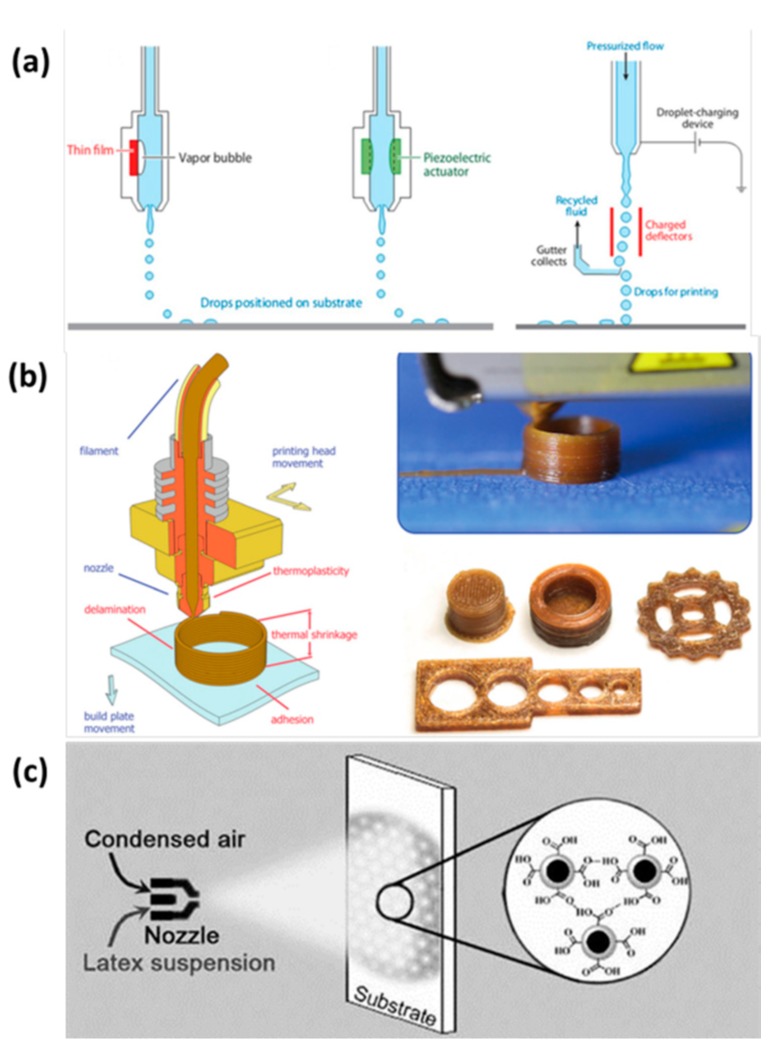
(**a**) Three typical processes of inkjet printing. Left: Thermal inkjet printing. Middle: Piezoelectric inkjet printing. Right: Continuous inkjet printing. Reproduced from [46], with permission from Annual Reviews, 2010; (**b**) Left: The process of extrusion printing technology. Right: Printing cylinder structure with the extrusion printing and the fabricated various structures. Reproduced from [48], with permission from Wiley, 2017; (**c**) Process of spray printing: ink spraying onto the substrate, sprayed layer self-assembling with a hydrogen bonding force. Reproduced from [50], with permission from Wiley, 2009.

The digital printing process is controlled by a computer signal, and the ink is deposited on the required place without a printing plate. The digital printing techniques could be divided into inkjet printing, extrusion printing, and spray printing. Inkjet printing is a directly printing technology, which uses the electrical signal to deposit functional ink without plate making. By virtue of the low-cost, convenience, flexibility, and speediness, inkjet printing has been widely used to fabricate high-quality patterns. As shown in Figure 3a, there are three typical processes of inkjet printing: thermal inkjet printing, piezoelectric inkjet printing, and continuous inkjet printing [46]. Inkjet printing has made important progress in fabricating images, photoelectric devices, and 3D structures. The depositing morphologies of inkjet printing could be controlled with substrate wettability, ink rheology, printing apparatus, and the external field. Many high-performance devices have been directly inkjet printed by adjusting the assembling structures of functional materials [47]. Recently, many 3D structures with various materials are fabricated by extrusion printing technology. The viscosity of ink could be very high with a large solid content. The technique could directly deposit the materials onto the substrate in an additive, non-contact, and reproducible manner. As shown in Figure 3b, the ink is extruded from the nozzle, and then the ink generates into a useful structure [48]. In order to fabricate high-resolution structures and devices, some precise extrusions are developed with special principles, such as electro-hydrodynamic printing and capillary force printing [49]. Spray printing is an efficient and speedy way to deposit various functional materials. The dispersed solution could be sprayed by spray gun with pressure or centrifugal force. The spray printing techniques could be divided into various types, such as air spray printing, electrostatic spray printing, thermal spray printing, automatic spray printing, low pressure spray printing, multi group spray printing, and so on. As shown in Figure 3c, the sprayed layer of functional materials could be adjusted to a uniform and fine structure with a self-assembling process [50]. The sprayed layer could also realize a pattern with the mask on the sprayed substrate.

## 3. Printable Nanomaterials in Fabricating Supercapacitors

Printing technology provides a valuable and effective way to pattern functional materials into high-performance micro-nano structures. A supercapacitor is composed of an electrode and electrolyte, in which the functional nanomaterials and assembled structures directly determine the performance in various areas of application. To fabricate high-performance supercapacitors, the printable materials should be dispersed into nanoscale. Therefore, many printable nanomaterials are studied in developing high-performance supercapacitors. To enhance the stability of the printed structure, the composite ink could be used for improving the mechanical strength. Meanwhile, the depositing structures could be precisely controlled by adjusting the coffee ring effect, evaporation or post-processing. These methods would enhance the stability of the printed structures. In the following, various nanomaterials used to fabricate supercapacitors by printing technology are presented.

### 3.1. Carbon Materials

Carbon material is an ideal electrode material for an electric double layer supercapacitor. Carbon nanomaterials are very common and readily available, so carbon materials are relatively inexpensive. Most importantly, carbon materials have a very large surface area and high electrical conductivity, which is very suitable for using in electric double layer supercapacitors. Meanwhile, the carbon material generally has porous characteristics, which facilitates the penetration of electrolyte into electrode material and increases the capacitance of the supercapacitor. At present, the carbon materials are used in supercapacitors including activated carbon, graphene, carbon nanotubes, and carbon fibers.

#### 3.1.1. Activated Carbon

**Figure 4:**
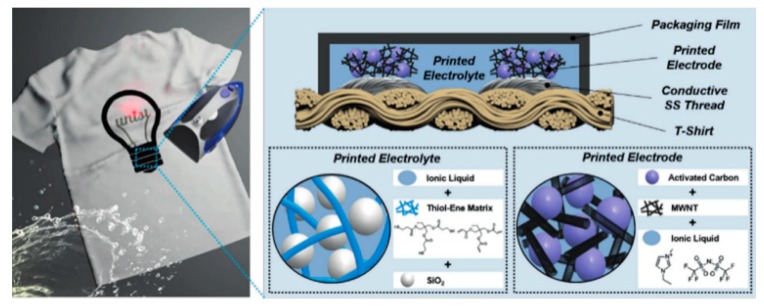
A schematic of screen printing activated carbon-based supercapacitors on clothes. **Left**: a supercapacitor printed on a T-shirt; **Right**: structure of a full-printed supercapacitor including electrodes and electrolytes. Reproduced from [54], with permission from Wiley, 2018.

**Figure 5:**
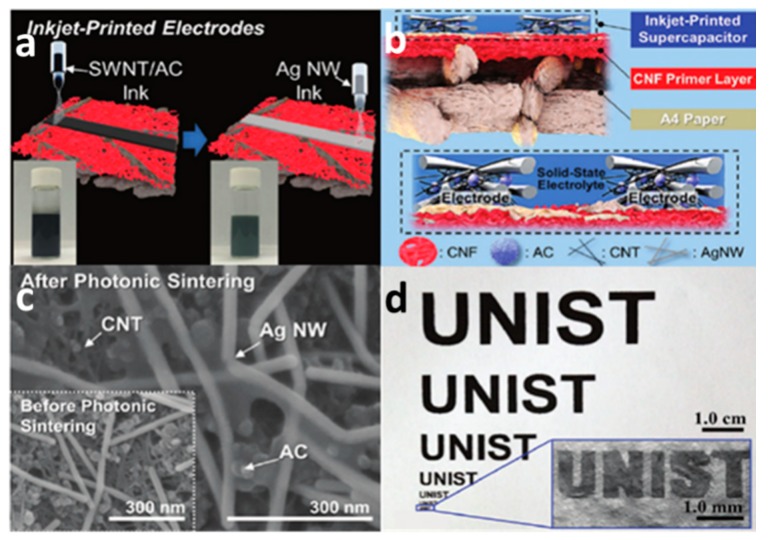
Schematic diagram of inkjet printing a supercapacitor with active carbon composite ink; (**a**) Prepare supercapacitors by inkjet printing AC/SWNT inks and Ag inks, respectively; (**b**) Inkjet printing AC/SWNT electrode structure; (**c**) Scanning electron microscope (SEM) images of inkjet printed AC/SWNT electrode; (**d**) Inkjet printed electrode with the shape of UNIST on A4 paper. Reproduced from [55], with permission from Royal Society of Chemistry, 2016.

Activated carbon is the most available carbon nanomaterial as it can be obtained from many biomass materials. J.H. Hou et al. successfully manufactured popcorn derived porous activated carbon tablets from corn popcorn using corn biomass [51], which has a high specific surface area (SBET: 3301 m^2^ g^−1^) and a high content of micropores (95% of micropore surface area, especially optimized for sub nanopore with a size of 0.69 nm). When the activated carbon in the ionic liquid electrolyzes, the assembled supercapacitor has an energy density of 103 Wh kg^−1^. In this work, the activated carbon shows the advantages of low cost, high specific surface area, and electrochemical stability. Moreover, activated carbon has a rich source of raw materials. In addition to coal, many biomass materials can be activated by physical or chemical activation to obtain activated carbon. Therefore, many researchers have used activated carbon solution as ink to print supercapacitors. S.Y. Lu et al. heated the rosettes at a high temperature and used H_2_O_2_, HAc to prepare graphene-like activated carbon nanoparticles with high electrical conductivity (0.81 Ω of the direct-current resistances at about 10 kHz frequency), large surface area (1014 m^2^ g^−1^), and high specific capacitance (340 F g^−1^ at 0.5 A g^−1^) [52]. The sheets were coated with activated carbon nanosheets and assembled into supercapacitors. The supercapacitors had a high specific energy density (23.33 to 16.67 Wh kg^−1^) and long cycle stability (98% after 10,000 cycles). The excellent properties of activated carbon could be obtained from different raw materials and preparing processes. The properties of activated carbon, such as pore structure, specific surface area, and surface functional groups, would play an important role in promoting the performance of the supercapacitor [53]. Pore structure would facilitate the storage of charge on the electrode surface in an electric double-layer structure. Meanwhile the surface functional groups would be beneficial to the oxidation–reduction reaction in the pseudocapacitor behavior. Thereby the pore structure improves the performance of the supercapacitor. In recent years, many researchers have studied activated carbon for the fabrication of high-performance supercapacitors by controlling pore size distribution, increasing specific surface area, and modifying the surface. As shown in the Figure 4, S.S. Lee et al. printed activated carbon ink on clothes by screen printing to prepare a flexible supercapacitor, which could maintain its electrochemical activity in the state of washing, wringing, and folding [54]. In addition, K.H. Choi et al. inkjet printed a high-resolution supercapacitor (Figure 5) on A4 paper using activated carbon/single-walled carbon nanotube (AC/SWNT) composite ink. The full-printed supercapacitor showed a high area capacitance of 100 μF cm^−2^ and good flexibility [55].

#### 3.1.2. Graphene

**Figure 6:**
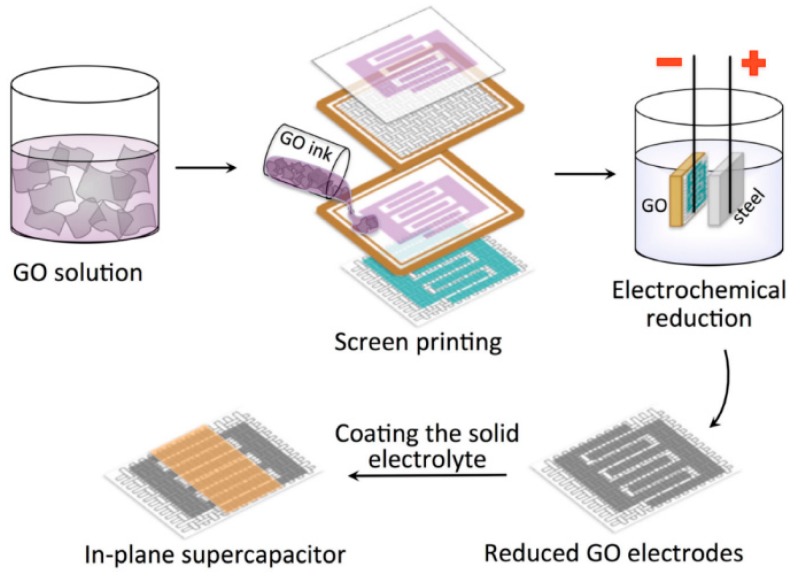
Screen printing of graphene oxide (GO) ink on fabrics and electrochemical reduction of graphene-based planar supercapacitors. Reproduced form [57], with permission from IOP Publishing Ltd., 2017.

**Figure 7:**
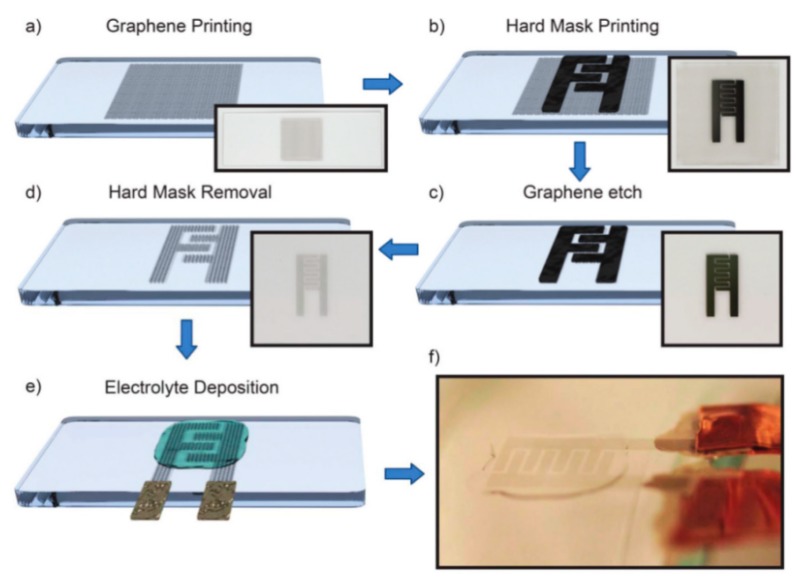
A schematic of a transparent type supercapacitor prepared by inkjet printing graphene ink. (**a**) Printing of graphene ink on a substrate; (**b**) Screen printing of silver ink as a current collector; (**c**) Plasma etching; (**d**) Removal of a mask; (**e**) Dripping the gel electrolyte; (**f**) Photographs of printed supercapacitors. Reproduced from [58], with permission from Royal Society of Chemistry, 2017.

**Figure 8:**
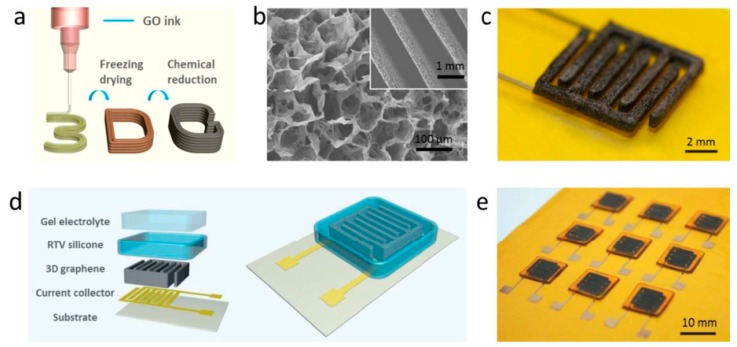
Schematic of printing a 3D micro supercapacitor. (**a**) 3D printing GO ink; (**b**) SEM image of 3D printed graphene electrode; (**c**) A 3D interdigitated structure of the electrode; (**d**) Assembling a 3D micro supercapacitor; (**e**) An array of the printed 3D micro supercapacitors. Reproduced from [60], with permission from Royal Society of Chemistry, 2013.

Graphene is a honeycomb planar film formed by sp2 hybridization of carbon atoms, which is a quasi-two-dimensional carbon nanomaterial with only one atomic layer thickness. Graphene has excellent optical, electrical, and mechanical properties, and has important application prospects in energy, micro-nano processing, materials science, biomedicine, and drug delivery, which is considered to be a revolutionary material in the future. Therefore, it is also called “black gold” and the “king of new materials”. Like activated carbon, graphene also has a high specific surface area (2630 m^2^ g^−1^ theoretically). Meanwhile, graphene has a high conductivity and excellent electrochemical activity. It is reported that the intrinsic capacitance of single layer graphene is about 21 mF cm^−2^. If the surface area of graphene is fully utilized, supercapacitors with graphene as the electrode material could harvest electric double layer capacitance values as high as 550 F g^−1^ [56]. Therefore, graphene has been the most widely used supercapacitor electrode material. As shown in Figure 6, A. M. Abdelkader et al. fabricated a solid state flexible supercapacitor on textiles by screen printing with graphene oxide ink, and the graphene oxide was reduced in situ by a rapid electrochemical method. Due to the strong interaction between the ink and the textile substrate, the electrodes exhibited excellent mechanical stability. The resulting supercapacitor exhibited excellent cycle stability at 10,000 cycles and maintained excellent mechanical flexibility [57]. In addition, D.S. Sollami et al. studied graphene ink for preparing a transparent, flexible supercapacitor by inkjet printing (Figure 7), which has a light transmittance of 71% and an area capacitance of 99 μF cm^2^ [58]. As planar graphene is prone to nano-material agglomeration, the actual measured capacitance of graphene-based supercapacitors is far below the theoretical value. Many researchers have combined graphene with other conductive materials to inhibit the agglomeration of graphene for improving the conductivity or capacitance of the composite. Y.Q. Liu et al. studied graphene oxide (GO)/polyaniline (PANI) composite ink for micro supercapacitors by extrusion printing, and the supercapacitor showed that the voltage window could be widened from 0.8 to 1.2 V and improvements could be achieved in energy density (from 3.36 to 4.83 mWh cm^−3^) and power density (from 9.82 to 25.3 W cm^−3^) [59]. The graphene could also be used as ink for fabricating a 3D structure. As shown in Figure 8, W.B. Li et al. studied graphene ink to prepare a 3D flexible micro supercapacitor through extrusion printing [60]. The devices demonstrated superior specific capacitance, an ultra-long circle life, stable mechanical flexibility, and high ability to integrate with microelectronics, which have promising prospects in portable and wearable electronics.

#### 3.1.3. Carbon Nanotubes

**Figure 9:**
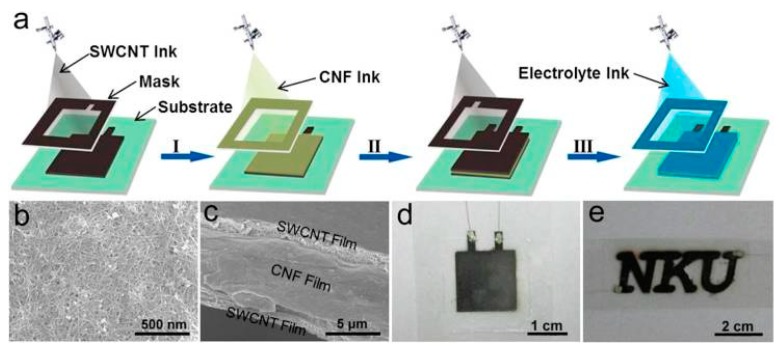
Carbon nanotubes (CNTs) ink was used to prepare a full-print supercapacitor by continuous spray printing. (**a**) Process of preparing supercapacitors by continuous spray printing with CNTs ink and electrolyte ink through a template; (**b**) SEM image of the spray printed CNTs; (**c**) Cross-sectional SEM image of fully printed CNTs electrode of supercapacitor; (**d**) A supercapacitor with the sandwich sturature; (**e**) Optical image supercapacitor with the shape of NKU. Reproduced from [62], with permission from American Chemical Society, 2017.

**Figure 10:**
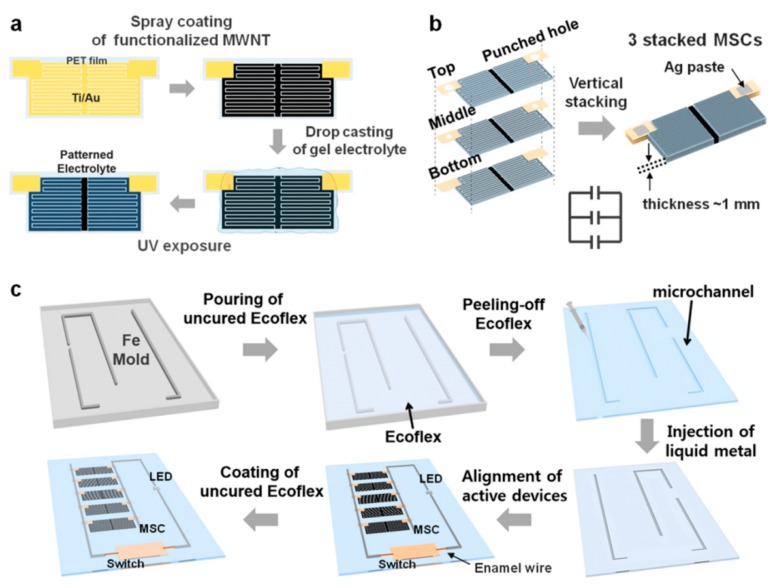
Schematic diagram of spray printing CNTs ink to prepare the micro supercapacitors. (**a**) Sprayed a micro-supercapacitor on the substrate; (**b**) Stack of three spray printed micro supercapacitors; (**c**) Spray printing a micro supercapacitor array on the substrate. Reproduced from [63], with permission from American Chemical Society, 2016.

Carbon nanotubes (CNTs), also known as Barker tubes, are one dimensional quantum materials with a special structure (radial size in the order of nanometers, axial dimensions in the order of micrometers, and tube ends are essentially sealed). The CNTs are usually multi-wall structures, which consist of several hundred to tens of layers of coaxial tubes composed of hexagonal arranged carbon atoms. The distance between the layers is fixed with about 0.34 nm, and the diameter is generally 2–20 nm. CNTs have many excellent properties such as heat resistance, corrosion resistance, and thermal conductivity. In addition, CNTs have excellent mechanical, electrical, optical, and chemical properties with the unique structural characteristics. Meanwhile CNTs have a large specific surface area, high electrical conductivity, and many chemical reaction sites [61]. Therefore, CNTs are studied in many applications as electrode materials of supercapacitors. As shown in Figure 9, X.Y. Wang et al. developed a continuous spraying strategy to fabricate supercapacitors with CNTs ink [62]. They fabricated flexible supercapacitors by printing CNTs inks on different substrates through screen printing and spray printing. H.J. Kim et al. produced a sealed, high-performance, scalable array of stacked planar micro supercapacitors as a wearable energy storage device by spray printing with CNTs ink for waterproof applications (Figure 10) [63].

#### 3.1.4. Carbon Fiber

Carbon fiber is a kind of carbon material with fiber morphology, carbon content higher than 90%, and excellent performance. It has a graphite microcrystalline structure and is composed of a graphite carbon layer oriented inside the fiber axis. Carbon fibers have outstanding mechanical properties including high tensile strength and modulus, low specific gravity, and outstanding electrical and thermal conductivity properties [64]. In addition, the carbon fiber pores are open, and the large, medium, and small pores are tightly connected, which is very favorable for electrolyte transport and charge adsorption. Carbon fiber also has excellent heat resistance, low swelling, and good chemical stability. Therefore, it could be an excellent electrode material for the fabrication of supercapacitors. J.C. Noh et al. combined carbon fiber with MnO_2_ to prepare a supercapacitor, which had an area capacitance of 4.86 mF cm^2^ and a wide operating voltage window of 2.0 V, and the electrodes exhibited excellent mechanical strength [65]. In practical applications, carbon fibers usually have poor electrochemical performance as electrode materials with their dense structure, low porosity, and small specific surface area (<10 m^2^ g^−1^). Therefore, the surface should be activated or treated to increase its specific surface area and improve its electrochemical performance.

### 3.2. Transition Metal Carbides/Carbonitrides or Nitrides (MXenes)

**Figure 11:**
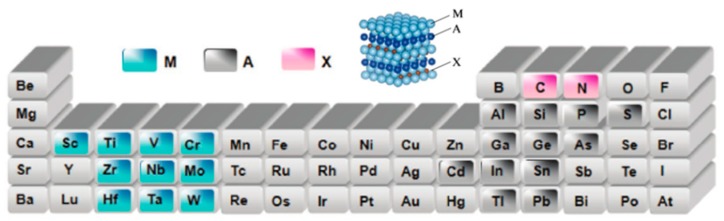
The possible constituent atoms of MAX in the element table. Reproduced from [66], with permission from Wiley, 2018. Stereogram of the 3D MAX phase. Reproduced from [70], with permission from Russian Academy of Sciences and Turpion Ltd., 2013.

Transition metal carbides/carbonitrides or nitrides (MXenes) are new members of the 2D material world. The material is typically produced by selectively etching the A layer from its MAX layer counterpart, which is a layered ternary carbide or a nitride with a formulation denoted as M_n+1_AX_n_ (*n* = 1–3), where M represents an early transition metal (such as Sc, Ti, Zr, V, Nb, Cr, or Mo), A is generally from the group IIIA or IVA (i.e., Group 13 or 14), and X is carbon and/or nitrogen. In 2011, Y. Gogotsi and his colleagues firstly reported the use of hydrofluoric acid (HF) for Al etching of Ti_3_AlC_2_ to produce graphene-like Ti_3_C_2_ MXene [66]. In this process, the surface of the Ti_3_C_2_ monolayer chemically terminates at O, OH, and/or F atoms. Following similar experimental procedures, the scientists designed the MXenes family, including Ti_2_C, Nb_2_C, Ti_3_CN, and so on, by using a corresponding MAX phase stripping of chemicals such as hydrofluoric acid, ammonium difluoride (NH_4_HF_2_), and so on [67]. There are two forms of MXenes with different M: solid solutions and ordered phases. In a solid solution, random arrangements of two different transition metals are observed in the M layer. In contrast, in the ordered phase, a single or double layer of a transition metal is sandwiched between layers of the second transition metal in the 2D carbide structure [68]. The researchers demonstrate that ordered MXenes are more energy-stable than solid solutions through computational density functional theory (DFT) for certain combinations of transition metals [69]. Presumably the possible constituent atoms of MAX are shown in Figure 11 [70]. It is predicted that there are more than 25 different MXenes.

Due to their metallic conductivity, high density, and hydrophilic nature, MXenes have proven to be promising candidates for supercapacitors with a high volumetric capacitance exceeding most previously reported materials [71,72]. For instance, titanium carbide (Ti_3_C_2_T_x_) clay films exhibits a high volumetric capacitance up to 900 F cm^−3^ with excellent cyclability and rate performance [73]. In addition, the gravimetric capacitance still needs to be enhanced. Generally, electrodes made of layered materials suffer from limited electrolyte-accessible surface area due to the restacking of the 2D sheets. This problem could be circumvented by constructing more open structures of layered materials that may provide more gallery space for storage and transport of electrolyte ions. Meanwhile, many research groups have done a lot of studies in MXenes, using as the super belt containers for the fabrication of flexible supercapacitors [74,75,76].

**Figure 12:**
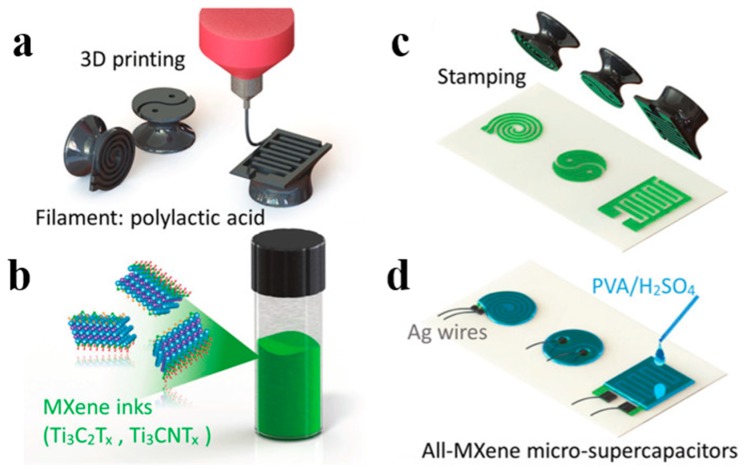
Fabricating the all-MXene micro supercapacitors with imprinting technique. (**a**) 3D printing process to create different shapes of seals; (**b**) Mxene inks; (**c**) Imprinting process; (**d**) All-Mxene micro supercapacitors. Reproduced from [77], with permission from Wiley, 2018.

In recent studies, many researchers studied the fabrication of supercapacitors by printing techniques with MXenes ink. C.F. Zhang et al. prepared 2D Ti_3_C_2_T_x_ ink for fabricating micro supercapacitors through a simple and low-cost imprinting method [77]. To verify the feasibility of this method, the researchers printed various shapes with the stamps (Figure 12a), coated the stamp with the prepared MXene ink (Figure 12b), and then pressed firmly on the paper substrate (Figure 12c,d). The cost and time of producing micro supercapacitors in this way was much lower and shorter than other methods such as inkjet printing and extrusion printing, which generally require specially processed substrates or complicated equipment and procedures. Meanwhile these solid-state Ti_3_C_2_T_x_ MXene micro supercapacitors exhibit an area capacitance of up to 61 mF cm^−2^ (C/A), long cycle lifetime, high energy density, and power density.

**Figure 13:**
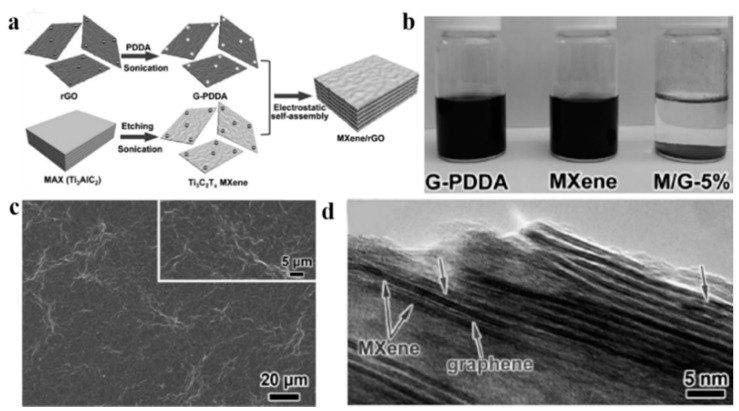
(**a**) Assembling a film with negatively charged MXene nanosheets and positively charged reduced graphene oxide (rGO) nanosheets; (**b**) Polymer poly(diallyldimethyl ammonium chloride)-modified rGO (G-PDDA) ink, Mxene ink, and the stable composite solution; (**c**) The obvious folds of composite film, indicating the supporting role of MXene nanosheets; (**d**) A good layered structure of the composite membrane, and the stacking of graphene oxide nanoplatelets was effectively prevented by the MXene nanosheet. Reproduced from [78], with permission from Wiley, 2017.

J. Yan et al. studied the fabrication of a flexible and highly conductive MXene/reduced graphene oxide (rGO) film by electrostatically self-assembling negatively charged MXene nanosheets and positively charged rGO nanosheets for ultra-fast supercapacitors (Figure 13a) [78]. Figure 13b shows the prepared ink. The obtained MXene/rGO composite could effectively relieve the self-capture of rGO and MXene, and maintain an ultra-high conductivity (2261 S cm^−1^) and high density (3.1 g cm^−3^). Figure 13c illustrates that MXene nanosheets played a very good supporting role. The presence of rGO nanosheets between MXene nanosheets could act as conductive spacers, which would increase the inter-lamellar spacing of MXene to provide unimpeded channels for electrolyte ions, and ensure high-speed ion transformation performance (Figure 13d).

To control the interlayer spacing of MXenes, J.M. Luo et al. obtained inspiration from the interesting structure of columnar layered clays, attempting to prepare pillared Ti_3_C_2_ MXene by simple liquid phase cetyltrimethylammonium bromide (CTAB) pre-dipping and Sn^4+^ columnization [79]. Based on the size of the intercalation precyclizer (cationic surfactant), the interlayer spacing of Ti_3_C_2_ MXene could be controlled and reached 2.708 nm, an increase of 177% compared to the original pitch of 0.977 nm. It is the largest value that we know. Due to the pillar effect, the assembled lithium-ion capacitors (LiCs) exhibited excellent energy density of 239.50 Wh kg^−1^ based on the weight of CTAB-Sn (IV) @Ti_3_C_2_ even at a higher power density of 10.8 kW kg^−1^. When the CTAB-Sn (IV) @Ti_3_C_2_ anode was combined with a commercial AC cathode, LiC showed higher energy density and power density than conventional MXene materials.

### 3.3. Conductive Polymers

In 1977, it was discovered that the electrical conductivity of polyacetylene films increased by nine orders of magnitude [80]. The discovery of this research broke the traditional view that organic polymers are all insulators, which opened the research field of conductive polymers and induced a worldwide research boom in conductive polymers. Many studies show that various conjugated polymers could be transformed into conductive polymers with different conductive properties after doping. Representative conjugated polymers are polyacetylene (PA) [81], polypyrrole (PPy) [82], polyaniline (PANI) [83], and polythiophene (PTi) [84], and so on.

In recent studies, conductive polymers have been an important electrode material for fabricating supercapacitors. The energy storage mechanism of conductive polymers is based on rapid and reversible type-and-type doping and dedoping on polymer electrodes, resulting in Faraday Eagle capacitors. The structure of the conductive polymer is an amorphous network structure with a wide gap between the molecules, which could accommodate a large number of large diameter anions or cations in the gap. This gap exists not only on the surface of the material but also inside the material, which allows the polymer to achieve a very high charge storage density and results in a very high capacitance. The outstanding advantages of conductive polymers are good electrical and optical properties, flexible mechanical properties, the unique solution processability, as well as electrochemical redox activity [85,86,87,88]. These excellent properties make conductive polymers have a wide range of applications in supercapacitors. In recent years, conductive polymers have made great progress in fabricating supercapacitors. Among them, nano-sized conductive polymers have attracted great attention for the beneficial effect on the performance of energy density. Therefore many research works have focused on the preparation and electrochemical behaviors of conductive polymers with nanostructures [89,90,91].

**Figure 14:**
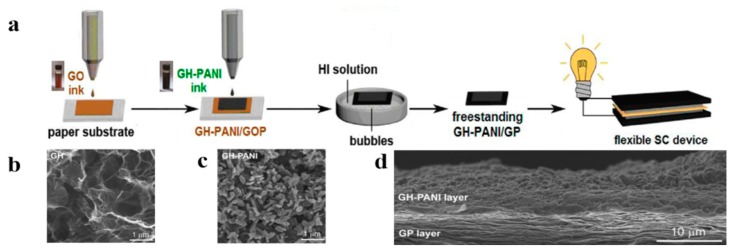
(**a**) Fully printed process for making a paper-based supercapacitor; (**b**) Graphene hydrogel (GH) with a porous network; (**c**) Coral-like nano-polyaniline (PANI) grown on graphene hydrogels; (**d**) Stacked stacks of graphene paper and nanocomposites. Reproduced from [92], with permission from American Chemical Society, 2014.

K. Chi et al. inkjet printed synthetic graphene hydrogel (GH) with PANI nanocomposites on the preprinted graphene paper (GP) [92]. Figure 14a shows the whole process of inkjet printing paper-based supercapacitor. Nano-polyaniline was grown on the GH scaffolds to improve the electrical conductivity, specific capacitance, and cycle stability of the nanocomposite (Figure 14b,c). With the π–π stacking action, the contact between the GP and nanocomposite material was promoted (Figure 14d). The flexible supercapacitor with the paper-based electrode demonstrated a superior performance with a maximum energy density of 24.02 Wh kg^−1^ at a power density of 400.33 W kg^−1^.

**Figure 15:**
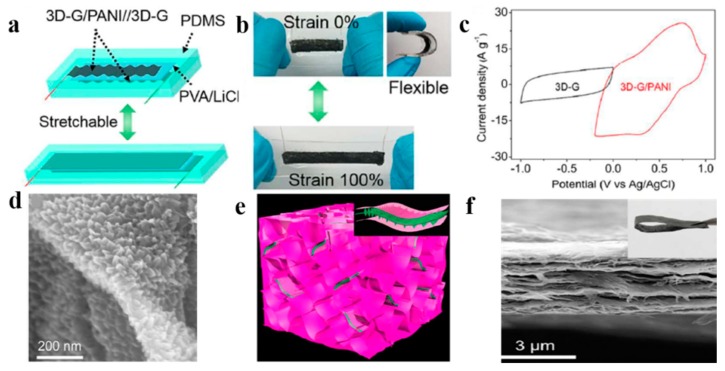
(**a**) Stretchable supercapacitors consisting of 3D graphene/polyaniline (3D-G/PANI) and 3D-G; (**b**) Good stretchability of the supercapacitor; (**c**) The 3D-G/PAN film shows an excellent capacitance performance; (**d**) Morphology of GO/PANI composite nanosheets with the grown PANI; (**e**) A 3D schematic of the GO/PANI nanomaterial embedded in the graphene skeleton (the top right corner is a partial enlargement); (**f**) A side-by-side screenshot of the composite film. Reproduced from [93], with permission from Royal Society of Chemistry, 2018.

K. Li et al. reported a new type of high performance stretchable supercapacitor with a 3D graphene/polyaniline (3D-G/PANI) composite material as the electrode active layer (Figure 15a,b) [93]. The unique 3D-G/PANI complex was realized by embedding the in situ polymerized G/PANI composite nanosheets in a powerful 3D graphene framework. Figure 15c shows that the structure has excellent specific capacitance. The stretchable asymmetric supercapacitor (SACS) was assembled on a pre-stretched elastic substrate. The supercapacitor achieved a high energy density of 77.8 Wh Kg^−1^. Firstly, the superior performance was attributed to the ultrathin PANI nanocluster array grown in situ on the graphene sheet, which promoted electron transfer, increased the utilization of PANI, and relieved the mechanical stress during doping/dedoping (Figure 15d). Secondly, 3D-G/PANI reduced the re-stacking of graphene sheets, which was beneficial to improve electrochemical performance (Figure 15e,f). Therefore, this 3D framework of graphene played an important role in fabricating the high performance stretchable supercapacitor. In addition, J. Maeng et al. studied to create an additional sidewall surface area with an ion etching substrate, and then an active conductive polymer material grew on the sidewall surface area in the form of nanofibers [94]. The fabricated supercapacitor showed a higher area capacitance and energy density than previously reported all-solid-state flexible capacitors, and exhibited good cycle performance under the bending conditions. These excellent properties were attributed not only to the additional surface area, but also to the fact that the nano form of polyaniline facilitated the transport of electrons.

### 3.4. Transition Metal Oxides or Hydroxides

In general, transition metal oxides or hydroxides have a higher energy density than conventional carbon materials. Meanwhile transition metal oxides or hydroxides have more stable electrochemical properties than conductive polymers. They can not only generate electric double layer storage charging like carbon materials, but also react with electrolyte ions to produce a Faraday reaction. The metal oxide or hydroxide materials currently used in supercapacitors include oxides and hydroxides of metal elements such as antimony (Sb), ruthenium (Ru), cobalt (Co), nickel (Ni), and manganese (Mn).

#### 3.4.1. RuO_2_

**Figure 16:**
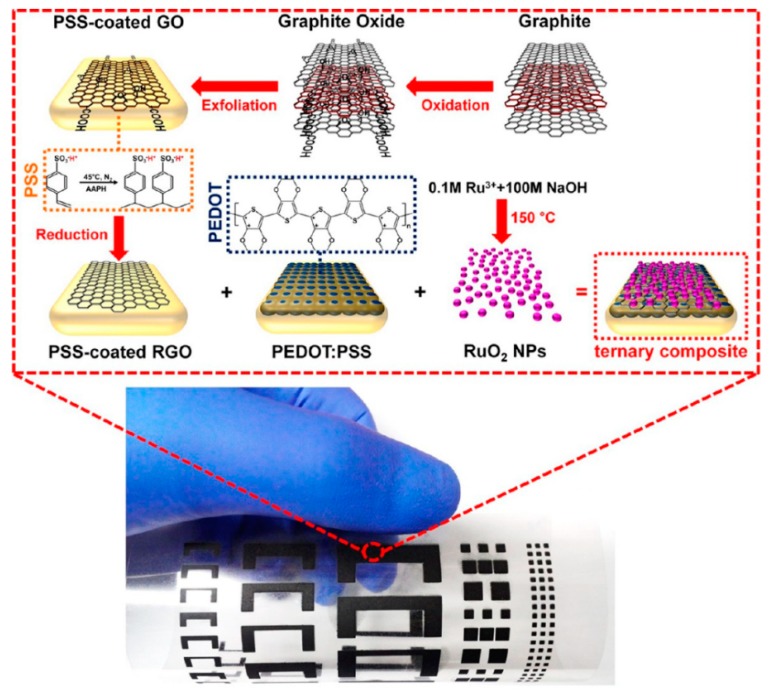
The whole process of screen printing the RuO_2_ composite electrode, and the sample of screen printed electrode of the supercapacitor. Reproduced from [96], with permission from American Chemical Society, 2015.

At present, RuO_2_ is the most common material studied among the metal oxide electrode materials of supercapacitors. RuO_2_ is the earliest transition metal oxide discovered and studied with pseudocapacitor behavior. The conductivity of metal oxides is generally poor, with the exception of RuO_2_ with high redox reversibility, high electrical conductivity, a wide potential window (up to 1.2 V), high theoretical specific capacity, good thermal stability, and a long cycle life, resulting in high energy density, power density, and cycle stability [95]. In addition, there are three different oxidation states within the voltage range of 1.2 V for Ru. During the charge–discharge process, the redox reaction is highly reversible between the three oxidation states. RuO_2_ could be presented stably in the aqueous electrolyte system of polyvinyl alcohol (PVA)/H_2_SO_4_. As shown in Figure 16, S.H. Cho et al. fabricated RuO_2_/Graphene/poly(3,4-ethylenedioxythiophene) polystyrene sulfonate (PEDOT:PSS) supercapacitor electrodes using screen printing; the resulting RuO_2_/PEDOT:PSS/Graphene electrode with a thickness of 5 μm exhibited a high conductivity (1570 S cm^−1^), a large specific capacitance (820 F g^−1^), and good cycling stability (81.5% after 1000 cycles) [96]. When RuO_2_ is used to produce the supercapacitors, the high cost and environmental harmfulness limit its application in commercial supercapacitors.

#### 3.4.2. MnO_2_

**Figure 17:**
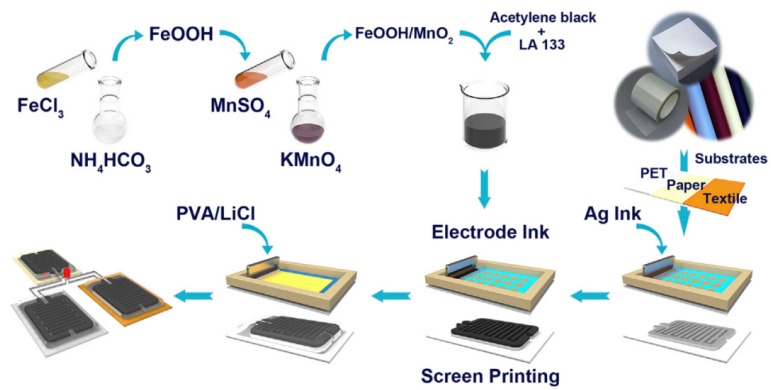
The preparation of MnO_2_ composite ink and the schematic diagram of screen printing MnO_2_ composite ink to prepare supercapacitor. Reproduced from [98], with permission from Elsevier, 2017.

**Figure 18:**
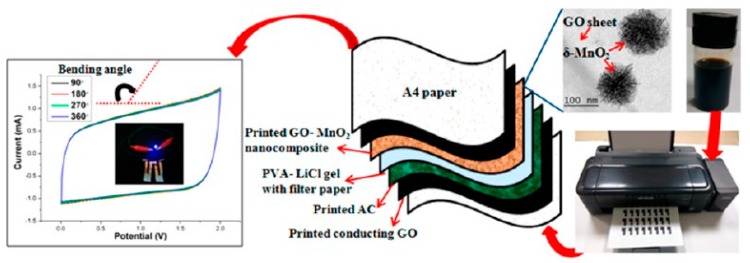
Preparation of supercapacitors by inkjet printing MnO_2_ ink, and characterization of its structure and properties. Reproduced from [99], with permission from American Chemical Society, 2017.

In recent years, cheaper and more environmentally friendly metal oxide electrode materials have received more and more attention from researchers, such as MnO_2_, NiO, V_2_O_5_, and Fe_3_O_4_. Among them, MnO_2_ was the most interesting metal oxide material due to its relatively low cost, low toxicity, environmental friendliness, and high theoretical capacity (1100–1300 F g^−1^). MnO_2_ is less expensive than noble metal oxides (RuO_2_, etc.), and it is one of the most valuable active materials with environmental friendliness and high theoretical specific capacitance (1370 F/g^−1^). In general, the conductivity of MnO_2_ (10^−5^–10^−6^ S/m) is very poor [97], which has become a major obstacle in the field of energy storage. In order to improve its conductivity, an effective method is compositing MnO_2_ with other conductive materials which act both as a current collector of the electrode and as a carrier of the MnO_2_. The energy storage mechanism of MnO_2_ is based on the adsorption and protonation of cations ion C^+^ (such as Na^+^, K^+^, etc.) in the electrolyte as the electrode material, and then a reversible oxidation–reduction reaction occurs to store the charge. As shown in Figure 17, Q. Lu et al. constructed an all-solid-state supercapacitor using MnO_2_/FeOOH by means of full screen printing technology, it exhibited a large specific capacitance (350.2 F g^−1^ at 0.5 A g^−1^), good rate capability (159.5 F g^−1^ at 20 A g^−1^), and outstanding cycling stability (95.6% capacitance retention after 10,000 cycles) [98]. In addition, P. Sundriyal et al. inkjet printed a solid state supercapacitor using MnO_2_ ink and a desktop printer, which had a high area capacitance of 1.586 F cm^2^ and a high specific capacitance of 1023 F g^−1^ (Figure 18) [99].

#### 3.4.3. NiO

With a large theoretical capacity, abundant resources, low cost, environmental friendliness, simple preparation process, easily accessible valence centers, nickel oxide (NiO) has also been widely studied as a cathode material for asymmetric supercapacitors [100]. Although NiO has many easily accessible valence states and excellent capacitance performance as a positive electrode material, its low hydrogen evolution potential in aqueous solution limits its application as a negative electrode material for asymmetric supercapacitors. G Meng et al. prepared a layered mesostructured NiO as the positive electrode of a supercapacitor. The specific capacitance of the electrode reached 3114 F g^−1^. The supercapacitor also showed a high energy density of 67 Wh Kg^−1^ and 1.6 V high voltage window ref. [101].

#### 3.4.4. Co(OH)_2_

When Co(OH)_2_ is involved in the charge and discharge of tantalum capacitors, Co^2+^ could be reversibly changed between bivalent (Co^2+^), trivalent (Co^3+^) and tetravalent (Co^4+^), thus providing a high theoretical capacity. Cobalt oxides or hydroxides have a very large theoretical pseudocapacitance of 3500–4600 F g^−1^ and a potential window of 0.45 V [102]. Different from RuO_2_ and MnO_2_, the crystal structure of Co(OH)_2_ also has a lamellar arrangement, so it could also be stripped into a layered nanosheet to obtain a larger specific surface area. Thereby it could generate a larger capacity. Xiaobo Ji et al. prepared supercapacitors by screen printing using Co(OH)_2_ inks, the Co(OH)_2_ asymmetric supercapacitor showed a maximum specific capacitance of 170 F g^−1^ at a current density of 0.5 μA g^−1^ in an aqueous electrolyte solution (3 M KOH) retaining 99.69% of its maximum capacity over 600 cycles [103].

### 3.5. Transition Metal Dichalcogenides

Transition metal dichalcogenides (TMDCs) are compounds of the formula MX_2_ wherein M is a transition metal element such as titanium (Ti), tantalum (Ta), molybdenum (Mo), and so on. X is sulfur (S), selenium (Se), chalcogenide (Te) and other chalcogens. These compounds all have a layered structure of X-M-X, and there are strong interactions between the particles in the layers, and adjacent sheets are stacked together by weaker van der Waals forces. According to the different relative positions of adjacent sheets, MX_2_ compounds could be divided into three different configurations of 1T (trigonal), 2H (hexagonal), and 3R (rhomboh) [104]. The similar structural characteristics of TMDCs and graphite make it easy to be stripped, forming a single layer of two-dimensional material or several layers of ultra-thin quasi-two-dimensional materials. Its large specific surface area and other characteristics also show considerable potential in the field of supercapacitors. The two-dimensional structure of the material could facilitate the intercalation and diffusion of ions in electrolyte. Meanwhile TMDCs could achieve high oxidation states in metal atoms of TMDCs, which is beneficial for improving the pseudocapacitance of supercapacitors. M. Liu et al. prepared an ultra-thin 1T′-MoTe_2_ nanosheet as an electrode for a supercapacitor, which had a high specific capacitance of 1393 F g^−1^ (current density at 1 A g^−1^) and the assembled supercapacitor also had a high energy density of 56.4 Wh kg^−1^ [105].

In order to clearly compare the advantages and disadvantages of each type of material, a comparison has been added as Table 1.

### 3.6. Printable Electrolyte

**Figure 19:**
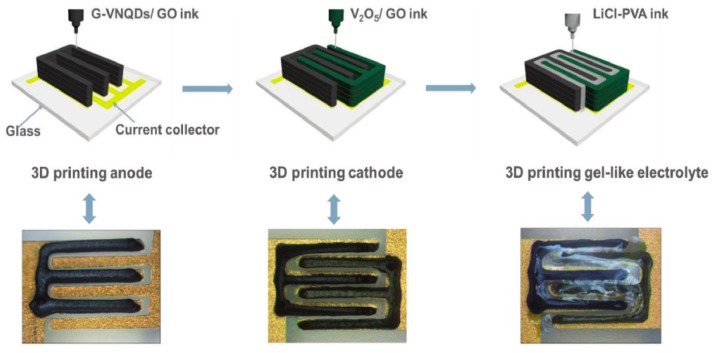
Schematic of a 3D printed micro supercapacitor. The GO composite ink was first printed as an electrode, then PVA/LiCl electrolyte was printed, and a full-printed supercapacitor was prepared. Reproduced from [107], with permission from Wiley, 2018.

Electrolyte is an important part of supercapacitors, and it is a bridge for charge diffusion in supercapacitors [106]. It plays a crucial role in the overall structure of the supercapacitor, such as bonding electrode particles, supplementing ions, and accelerating ion conduction. Electrolyte decomposition voltage, conductivity, and applicable temperature range are three important indicators for the application of the electrolyte. In addition, the electrolyte must have good wettability with the electrode material, so that the electrode could be contact fully with the electrolyte. It is well known that most electrolytes are liquid materials, which can be assembled into supercapacitors by printing, providing a good basis for full-printing supercapacitors. Electrolyte materials are classified into liquid electrolyte, solid electrolyte, and gel electrolyte. Each type of electrolyte has unique advantages. However, the leakage of liquid electrolytes and the slow transfer of solid electrolyte ions limit their use in energy storage devices such as supercapacitors. Therefore, gel electrolyte is widely used in fabricating supercapacitors. The physical property of the gel electrolyte determines that it could be assembled into supercapacitor by printing technology, which is useful for applying in full-printed supercapacitor. Therefore, the printable electrolyte is mostly the gel electrolyte. In the future, the solid electrolyte processing as a solution could also be used as ink to print supercapacitor. For example, Q. Lu et al. printed the PVA/LiCl gel electrolyte on the electrode material by screen printing, and a full-printed supercapacitor was assembled with electrolyte distributed on the electrode [98]. In addition, K. Shen et al. printed electrodes and PVA/LiCl electrolyte inks to fabricate supercapacitors by extrusion printing technique (Figure 19). The 3D printed asymmetric micro supercapacitor with the interdigitated electrode and electrolyte exhibited excellent structural integrity, an ultrahigh real capacitance of 207.9 mF cm^−2^, and a wide electrochemical potential window of 1.6 V [107]. The printable electrolyte would contribute to realizing full printed supercapacitors.

## 4. Conclusions and Outlook

In this paper, recent advances in printable nanomaterials for the fabrication of high-performance supercapacitors have been reviewed. The advantages of printing technology are summarized with traditional and emerging techniques. Moreover, various nanomaterials for the fabrication of supercapacitors by printing technology are presented, including carbon materials, transition metal carbides/carbonitrides or nitrides, conductive polymers, transition metal oxides or hydroxides, transition metal dichalcogenides, and electrolyte. On the basis of printability and electrochemical performance of nanomaterials, various high-performance supercapacitors have been fabricated by printing technology.

Although plenty of research has been devoted to printed supercapacitors with functional nanomaterials, there are still many challenges to realize high-performance supercapacitors in some areas of application. In detailed studies of the interactions between the evaporation of printed droplets and the structure of deposited nanomaterials, introducing external fields and special printing apparatuses will enhance the controllability of micro-nano structures of electrodes and electrolytes in supercapacitors. Moreover, theoretical research on the solute distribution in the coalesced droplets is necessary for printing precise 3D structure of electrode and electrolyte in supercapacitors. In addition, research on flexible and stretchable nanomaterials has become an advanced research hotspot in the field of wearable devices. The controllable assembly of nanomaterials during the evaporating process of printed droplets and effective composing of nanomaterials with different properties would provide a valuable route to fabricating portable and wearable supercapacitors. Therefore printable nanomaterials could produce excellent supercapacitor performance, and the promising applications of printed high-performance supercapacitors could be foreseen in the near future.

## Figures and Tables

**Table 1:** Comparing the advantages and disadvantages of each type of material.

	Feature	Advantage	Disadvantage
Material			
Carbon		Large specific surface area High conductivity Electrochemical stability Cheap price	Low energy density Poor dispersity
MXenes		Large specific surface area High conductivity Good dispersity Higher area capacitance	Complex production High prices Lower mass capacitance
Conductive polymers		High specific capacitance Unique solution processability Filming has good flexibility	Low conductivity Poor electrochemical stability
Transition metal oxides or hydroxides		High specific capacitance High energy density Wide potential window Low cost Easy to prepare	Low conductivity Poor electrochemical stability
Transition metal dichalcogenides		Large specific surface area High specific capacitance Good electrochemical stability	Preparing method is not mature. Physical and chemical properties are easily affected by the environment.
